# Nonoperative Management of Penetrating Right Thoracoabdominal Injury

**DOI:** 10.7759/cureus.15170

**Published:** 2021-05-22

**Authors:** Mohamed Ahmed, Amarseen Mikael, Yara Gorski, Ahmed Mahmoud, Raymund Cordero

**Affiliations:** 1 Surgery, University of California, Riverside, USA; 2 Surgery, Riverside Community Hospital, Riverside, USA; 3 Surgery, Temecula Valley Hospital, Temecula, USA

**Keywords:** nonoperative, gsw, trauma management, liver trauma, penetrating injuries

## Abstract

Penetrating thoracoabdominal injuries caused by stabbing or firearms are seen on an almost daily basis at trauma centers in the USA. The nonoperative management of carefully selected hemodynamically stable patients is still under dispute. We present a case of right thoracoabdominal firearm injury managed nonoperatively.

## Introduction

In the 1960s, operative management of gunshot wounds (GSWs) to the abdomen was the standard of care. The mantra was that all “GSWs of the abdomen should be explored as soon as the patient's condition permits" [1]. Two decades later, a prospective study concluded that 33% of patients with penetrating liver injuries were successfully managed nonoperatively [2]. While nonoperative management of blunt abdominal solid organ injuries is the standard of care, routine surgical exploration remains the current practice for penetrating solid organ injuries [3]. In 2019, a retrospective study concluded that selective nonoperative management was not only acceptable but also independently associated with improved survival and decreased complications [4].

## Case presentation

**Figure 1:**
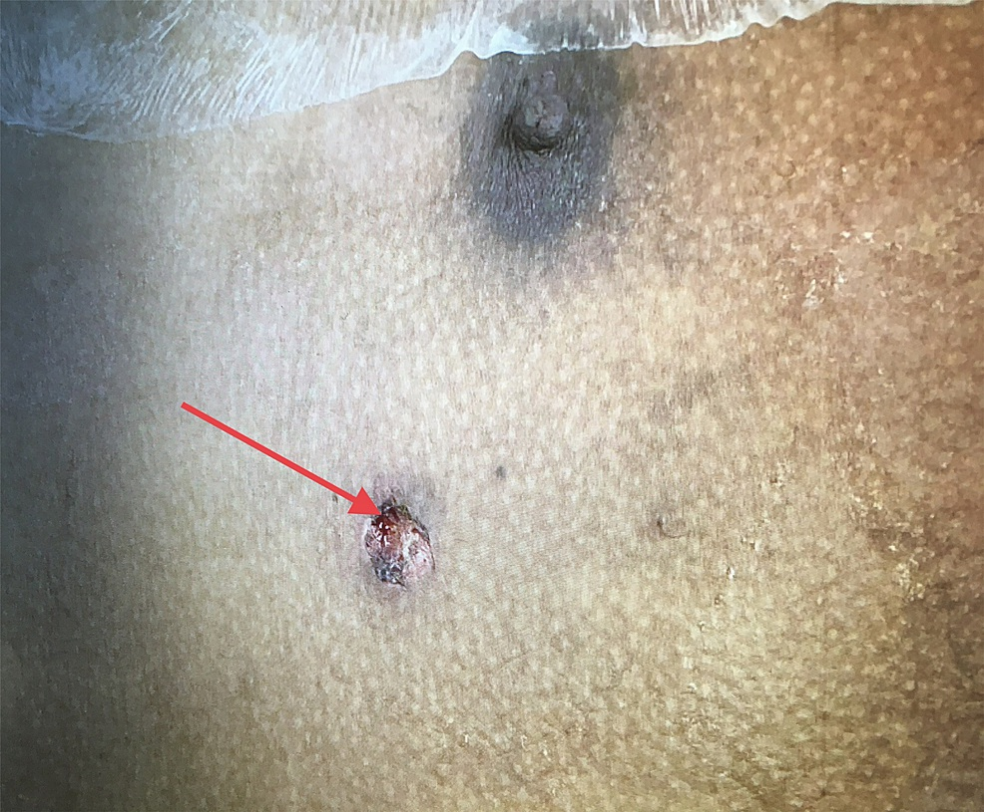
GSW to right chest below the nipple Red arrow shows the entry wound, below the right nipple concerning for thoracoabdominal injury

**Figure 2:**
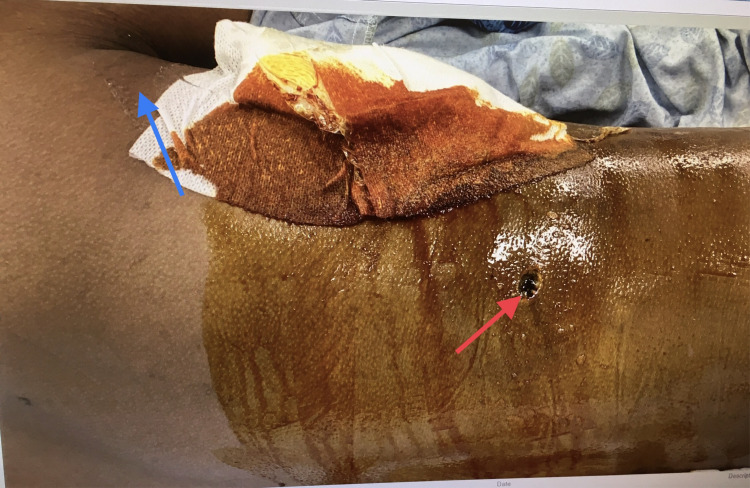
Exit wound Red arrow shows the exit wound in the right flank, and blue arrow shows the patient’s axilla.

A 23-year-old male patient was brought to our emergency room after sustaining a gunshot to the right lower chest (Figure 1) with an exit wound in the right flank (Figure 2).

**Figure 3:**
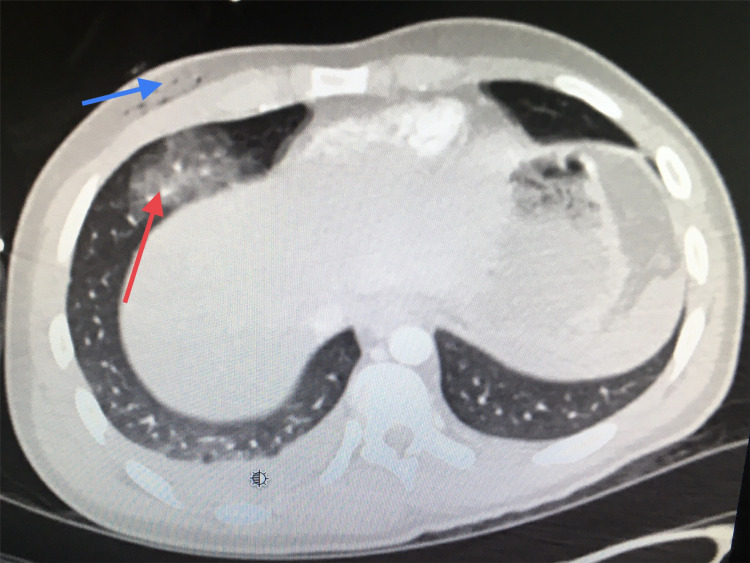
CT scan of the chest Red arrow shows lung laceration secondary to gunshot injury, and blue arrow shows subcutaneous air where the bullet traveled.

The patient was hemodynamically stable (blood pressure of 95/65 mmHg and pulse rate of 67 beats per minute) with an oxygen saturation of 100% on room air. The physical examination revealed an exit wound in the right flank area with a soft non-tender abdomen. Focused Assessment with Sonography for Trauma (FAST) scan was negative for intraperitoneal free fluid. Laboratory findings were within normal limits, except an elevated liver function test with alanine aminotransferase (ALT) at 1,172 U/L (normal range: 7-55 U/L) and aspartate transaminase (AST) at 3,657 U/L (normal range: 5-40 U/L). Given the clinical findings and the absence of any sign requiring exploratory laparotomy (peritonitis, hemodynamic instability, or evisceration), computed tomography (CT) was performed. CT of the chest revealed a right fifth rib fracture, ground-glass opacity at the right lung base concerning for lung laceration, a small hemopneumothorax, and subcutaneous emphysema within the anterior right chest wall (Figure 3).

**Figure 4:**
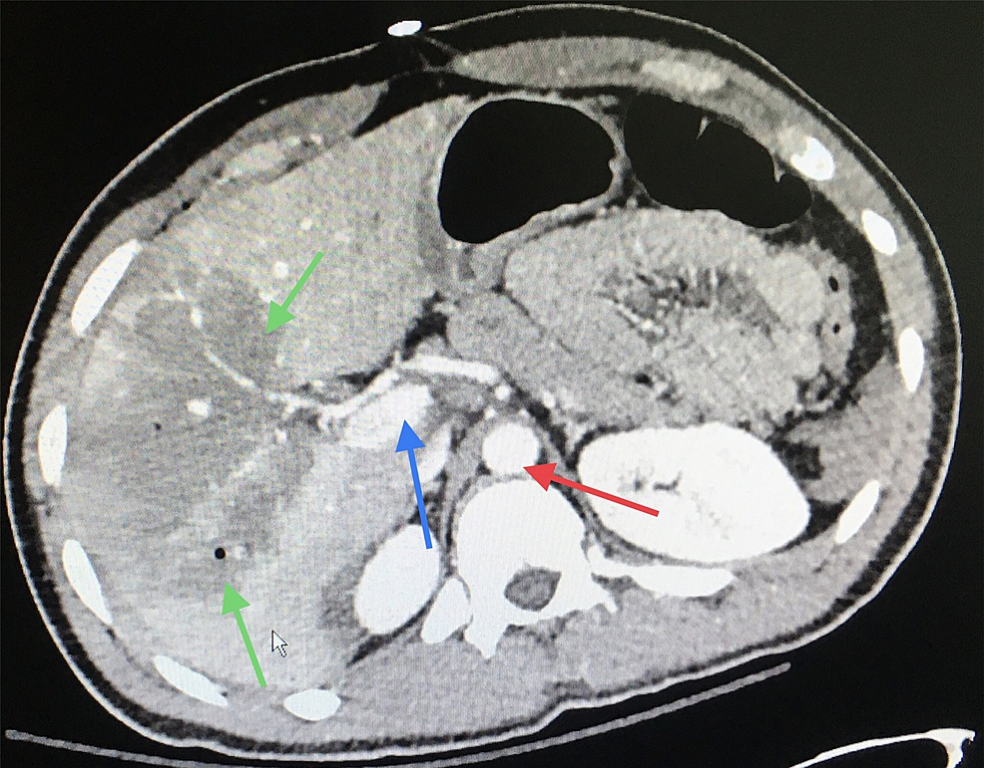
CT of the abdomen Green arrows show liver parenchymal disruption involving more than 25% of the right lobe with active bleeding (grade 4 liver injury), blue arrow shows extravasation of contrast consistent with active bleeding, and red arrow shows aorta color similar to the extravasation.

CT of the abdomen and pelvis revealed grade 4 liver injury with active extravasation and moderate hemoperitoneum (Figure 4).

**Figure 5:**
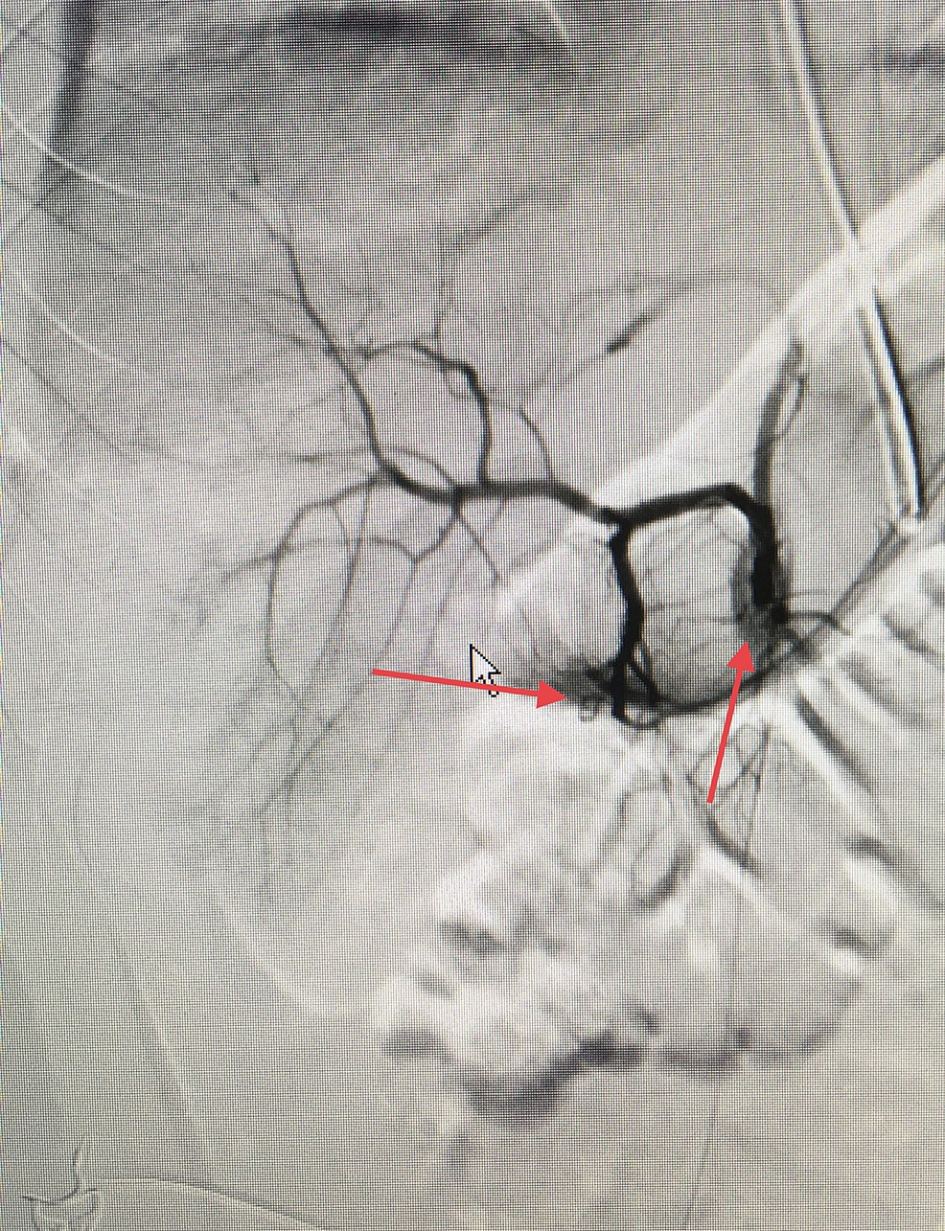
Angiography showing active extravasation; contrast is seen outside the vessel (red arrows)

The patient remained hemodynamically stable and was taken to our angiography suite. Angiography was performed, which revealed right hepatic artery extravasation (Figure 5).

**Figure 6:**
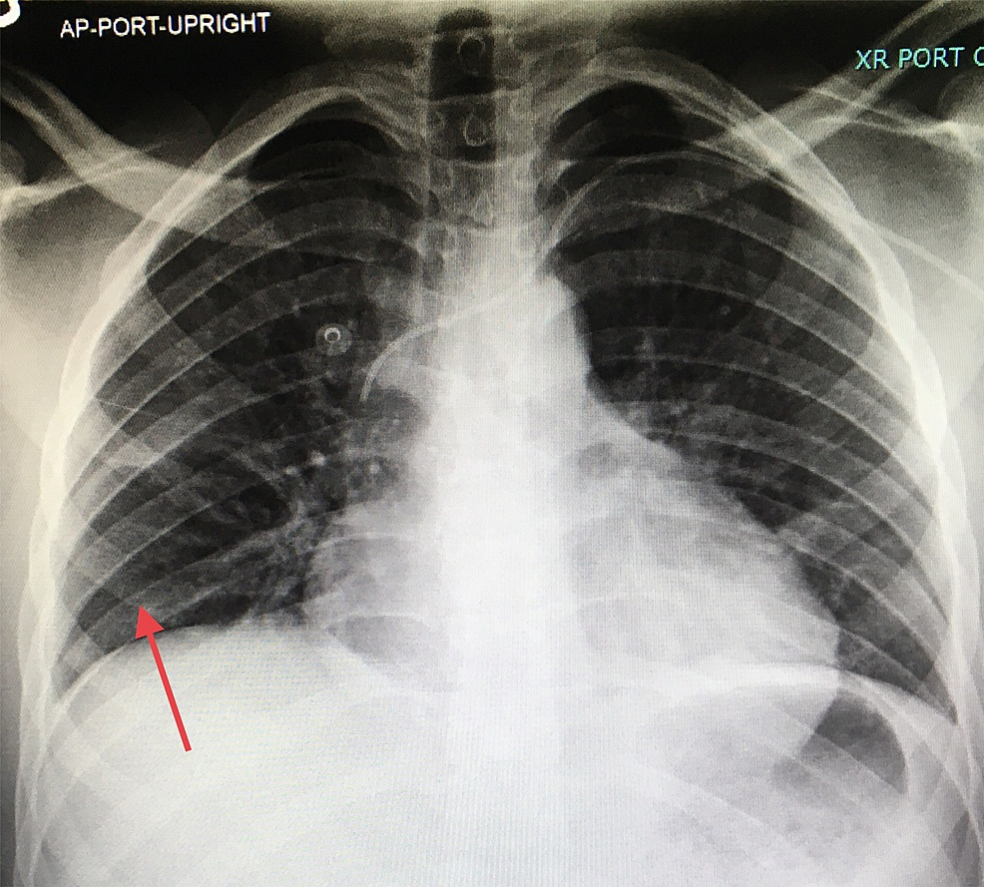
Chest X-ray Following chest tube removal, no hemothorax or pneumothorax was seen in the right chest (red arrow).

Successful Gelfoam embolization was performed. Right chest tube thoracostomy was performed and the hemothorax was drained. The patient remained hemodynamically stable with a soft non-tender abdomen over the following 24 hours of observation in our intensive care unit. The chest tube was removed on hospital day 8 (Figure 6), and the patient was discharged home.

The patient declined laparoscopy with the possible repair of the diaphragm during a follow-up visit.

## Discussion

The thoracoabdominal region is defined as the fourth intercostal space superiorly (nipple level) and the costal margin inferiorly (around the entire torso). This changes with the movement of the diaphragm as well. The management of penetrating torso trauma has seen repeated cycles involving both operative and nonoperative algorithms [5]. Similarly, the management of penetrating abdominal trauma went through many cycles until the 1960s when the concept of nonoperative management of selected penetrating abdominal wounds was reintroduced [6]. With more and more reports emphasizing the high complications rate (20%) of laparotomies in penetrating abdominal trauma without associated injuries and up to 33% negative laparotomy rate [7,8], selective nonoperative management of penetrating abdominal trauma has become increasingly popular for both stab and GSWs over the last two decades [9]. Patients presenting in extremis due to penetrating trauma to the thoracoabdominal region should undergo resuscitative thoracotomy emergently [10]. In unstable patients, the determination of which anatomic cavity to explore primarily becomes of utmost importance. Ultrasonography assists in excluding cardiac injury or intra-abdominal hemorrhage. It is important to keep in mind that many patients with solid-organ injuries do not require surgical intervention [11]. Penetrating abdominal trauma is commonly caused by a stab or gunshot, with the small bowel as the most frequently injured organ (50%) followed by the large bowel (40%), liver (30%), and intra-abdominal vascular injury (25%) [12]. In patients with no hollow viscous injury, vascular injury, or a clear indication for laparotomy, selective nonoperative management of thoracoabdominal penetrating injury might be associated with lower morbidity and a high success rate [13,14]. Currently, there is no consensus in regard to the repair of the right diaphragm injury and it is believed to have minimal consequences and "protection" from herniation by the large fixed liver, as supported by animal experimental studies with strong evidence of spontaneous healing in more than 90% of injuries [15,16].

## Conclusions

Selective nonoperative management of penetrating trauma has resulted in a paradigm shift away from routine trauma laparotomy and has reduced the number of unnecessary operations with its potential morbidity and complications. In the appropriate environment (close observation, serial clinical examinations), carefully selected penetrating thoracoabdominal injured patients with solid organ injury can be managed nonoperatively. While it is recommended to plan the repair of the left diaphragm injury, right diaphragm injury repair continues to be controversial.
